# FUNCTIONAL IMPROVEMENTS AMONG OLDER VETERANS WITH PAD FOLLOWING 12 MONTHS OF EXERCISE TRAINING

**DOI:** 10.1093/geroni/igac059.1634

**Published:** 2022-12-20

**Authors:** J Antonio Gutierrez, Megan Pearson, Lin Gu, Katherine Hall, Sunil Rao, Miriam Morey

**Affiliations:** Durham VA Medical Center, Durham, North Carolina, United States; Durham VA Healthcare System, Durham, North Carolina, United States; Durham VA Medical Center, Durham, North Carolina, United States; Durham VA Health Care System, Durham, North Carolina, United States; DURHAM VA MEDICAL CENTER, DURHAM, North Carolina, United States; Durham VA Health Care System, Durham, North Carolina, United States

## Abstract

The prevalence of peripheral artery disease (PAD) increases exponentially with age and is associated with heightened risk of functional impairment. We assessed baseline function and compared changes in mobility among older Veterans with PAD participating in Gerofit, a clinical exercise program. A total of 545 Veterans (mean age: 79 years) completed baseline, 3-, 6-, and 12-month mobility assessments and were divided into the following cohorts: PAD (n=99) and no PAD (n=446). Assessments included 10-m walk speed, 6-min walk distance, 30-s chair stands, and 8-ft up-and-go time. Veterans with PAD performed worse on all measures than non-PAD Veterans at baseline. Veterans with PAD demonstrated significant improvements (p < 0.05) in all measures from baseline at 3, 6 and 12 months. There were no significant differences in change scores between PAD and non PAD across all time points, controlling for age, except for 8-ft up-and go. PAD Veterans benefit from function-based exercise.

